# Probing Charge Transport Difference in Parallel and Vertical Layered Electronics with Thin Graphite Source/Drain Contacts

**DOI:** 10.1038/s41598-019-56576-8

**Published:** 2019-12-27

**Authors:** Jiayi Li, Ko-Chun Lee, Meng-Hsun Hsieh, Shih-Hsien Yang, Yuan-Ming Chang, Jen-Kuei Chang, Che-Yi Lin, Yen-Fu Lin

**Affiliations:** 1 College of Physics, Sichuan University, Chengdu, Sichuan 610064 China; 2 Department of Physics, National Chung Hsing University, Taichung, 40227 Taiwan; 3 Institute of Nanoscience, National Chung Hsing University, Taichung, 40227 Taiwan

**Keywords:** Electronic and spintronic devices, Electronic devices

## Abstract

In the present study, we aim to help improve the design of van der Waals stacking, i.e., vertical 2D electronics, by probing charge transport differences in both *parallel* and *vertical* conducting channels of layered molybdenum disulfide (MoS_2_), with thin graphite acting as source and drain electrodes. To avoid systematic errors and variable contact contributions to the MoS_2_ channel, parallel and vertical electronics are all fabricated and measured on the same conducting material. Large differences in the on/off current ratio, mobility, and charge fluctuations, between parallel and vertical electronics are evident in electrical performance as well as in charge transport mechanisms. Further insights are drawn from a well-constrained analysis of both temperature-dependent current-voltage characteristics and low-frequency (LF) current fluctuations. This work offers significant insight into the fundamental understanding of charge transport and the development of future layered-materials-based integration technology.

## Introduction

Integrated circuits (ICs) have dramatically changed people’s lives in the past few decades. Nowadays, almost all electronic products possess a high packing density with ICs of numerous functions. In this era of rapid development of ICs, electronics performance has made significant progress in terms of size, efficiency, and cost. Nevertheless, the development of next-generation electronics is a vital issue and has received widespread attention in recent years. With the 2004 discovery of graphene, an extended honeycomb network of single carbon atoms, research on two-dimensional (2D), layered electronics has opened a window for transcending the current limit of Moore’s Law^1^. Electronics constructed with channels layered in semiconducting transition metal dichalcogenides, such as MoS_2_, are among the most promising candidates, with related research showing substantial advances^2–6^. To develop reliable 2D electronics, the electrical mechanisms of layered materials in contact with metals or metal-like graphene have been studied^7–9^. Previous authors have already demonstrated the existence of a Schottky barrier at the interface of the metal and the layered material, where charge carriers overcome this barrier at higher temperatures and show thermally-assisted tunnelling at lower temperatures^7^. However, it has now been experimentally proven that the small work function difference between graphene and layered materials allows graphene as a transistor contact to reduce the contribution of contact resistance and then form an Ohmic contact, achieving superior device performance^10–14^. Recently, these transistors with layered conducting channels connected in-plane have been surpassed by van der Waals heterostructures, formed by the layer-by-layer integration of various 2D materials in the vertical direction. This development has opened a whole new class of materials in condensed matter physics, some of which are being recognized as building blocks for the framework of novel three-dimensional (3D) artificial structures^14–18^. Pioneering achievements using these vertically stacked heterostructures have been demonstrated in diverse applications, such as logic devices^19^, atomically thin p-n junctions^20^, van der Waals memristors^21^, tunnelling transistors^22^, and even multifunctional electronics^17,19,23^. Although atomically thin 2D electronics present rosy prospects for the future, we still lack a complete understanding of performance difference as well as charge transport for carrier passing through parallel/vertical conducting channels^24–27^.

In the present work, a layered MoS_2_ van der Waals heterostructure with thin graphite acting as contacting electrodes was designed and fabricated by mechanical exfoliation and dry-transfer methods. The unique configuration, with two drains and one source, can conduct through both parallel and vertical channels in the same MoS_2_ flake. This configuration thus provides insight into charge transport mechanisms and shows the differences in electrical properties between the different transport directions. We emphasize that the use of thin graphite as contacting electrodes reduces the impact of contact resistance on electronics and further exposes the electrical contribution of interlayer resistance between adjacent MoS_2_ layers in the vertical conducting channel. Through a careful analysis of temperature dependence of current-voltage (*I*_ds_ − *V*_ds_) behaviors in our layered MoS_2_ heterostructure, electrical performance was systematically compared between parallel and vertical channels. Unlike the electrical contribution from contact potential in the previous works^16,28^, here the electrical properties of charge transport are dominated mainly by the MoS_2_ conducting channels. We found that the operation of the MoS_2_ heterostructure under a drain-source voltage (*V*_ds_) of 25 mV was characterized by a current on/off modulation of ~10^6^ for parallel conducting channels but less than 10^2^ for vertical conducting channels. In addition, mobility variation with decreasing temperature displayed obvious upward and downward trends, respectively, for parallel and vertical electronics. This pattern suggests the domination of interlayer resistance in the vertical conducting channel. In addition to quasi-static measurements, dynamic current fluctuation measurements were also carried out to re-address the influence of interlayer resistance. Although a clear 1/*f* noise dependence was observed for both parallel and vertical channels, an additional resistor contribution was found for charge transport in the vertical direction, strongly indicating the existence of interlayer resistance. The MoS_2_ heterostructure used in this work offers a simple configuration to probe charge transport in both parallel and vertical conducting paths. The experimental observations not only provide a basic understanding of electrical properties for layered electronics, but also pave the way for using van der Waals heterostructures to develop future 3D artificial configurations.

## Results

### Device structures

**Figure 1:**
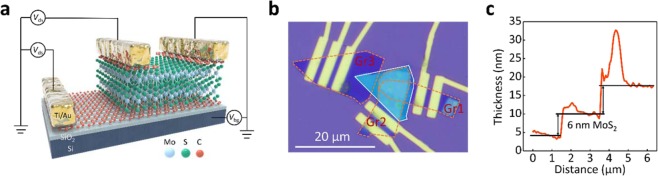
(**a**) Schematic diagram of the layered MoS_2_ van der Waals heterostructure with a circuit diagram overlaid. (**b**) Optical microscopic image of the MoS_2_ heterostructure, composed of three thin graphite (outlined in dashed orange lines), a MoS_2_ channel (outlined in solid white lines), and Ti/Au metal electrodes. (**c**) Typical thickness of the MoS_2_ conducting channel and thin graphite as measured by atomic force microscopy line profile.

Figure 1(a) schematically illustrates the parallel and vertical MoS_2_ electronics, stacked with one multilayer MoS_2_ channel and three thin graphite electrodes. Thanks to both the particular device configuration and the thin graphite composition of the electrodes, it is possible to ignore the contribution of contact resistance and to more precisely explore transport differences between parallel and vertical channels in the same device. To modulate the position of the MoS_2_ Fermi level as well as carrier concentrations, a heavily doped Si substrate was used as the back-gate electrode (*V*_bg_). Figure 1(b) shows an optical image of the van der Waals heterostructure device on a Si/SiO_2_ substrate. The shape of the MoS_2_ channel is outlined in solid white, while those of the thin graphite (Gr) electrodes are outlined in dashed orange. In the process of device fabrication, thin graphite 1 (Gr1) was first mechanically exfoliated and transferred on a heavily n-doped silicon substrate with a silicon oxide surface coating of 300 nm thickness; this surface served as the bottom electrode for the MoS_2_ channel. The MoS_2_ flake was then stacked on top of the Gr1 using the same technique. After that, thin graphite 2 (Gr2) and graphite 3 (Gr3) were sequentially stacked on the MoS_2_ channel without overlapping, acting as the source/drain electrodes for the parallel MoS_2_ channel. The overlapping portion of the Gr1/MoS_2_/Gr2 sandwich sets the position of the vertical electronics. For the purpose of characterizing electrical mechanisms, several outside pads were finally pattered on the top of Gr1, Gr2, and Gr3, using standard electron-beam lithography, followed by thermal evaporation of Ti/Au (5/50 nm thick). In this work, because of the limited density of states and weak electrostatic screening effect of thin graphite^16^, the MoS_2_ Fermi level should be effectively modulated through the back-gate electrode. Typical thicknesses of the MoS_2_ channel and thin graphite electrodes were further determined by atomic force microscopy (AFM). From the line profile of the AFM image, as shown in Fig. 1(c), the thickness of the MoS_2_ channel used in this work was about 6 nm, which is consistent with the height of a 9-layer MoS_2_ stack, while the thickness of thin graphite electrodes was determined to be 7 nm. Noticed that the thickness for Gr1, 2 and 3 is intentionally selected to be similar for making an easy comparison in electrical properties.

### Electrical characteristics

**Figure 2:**
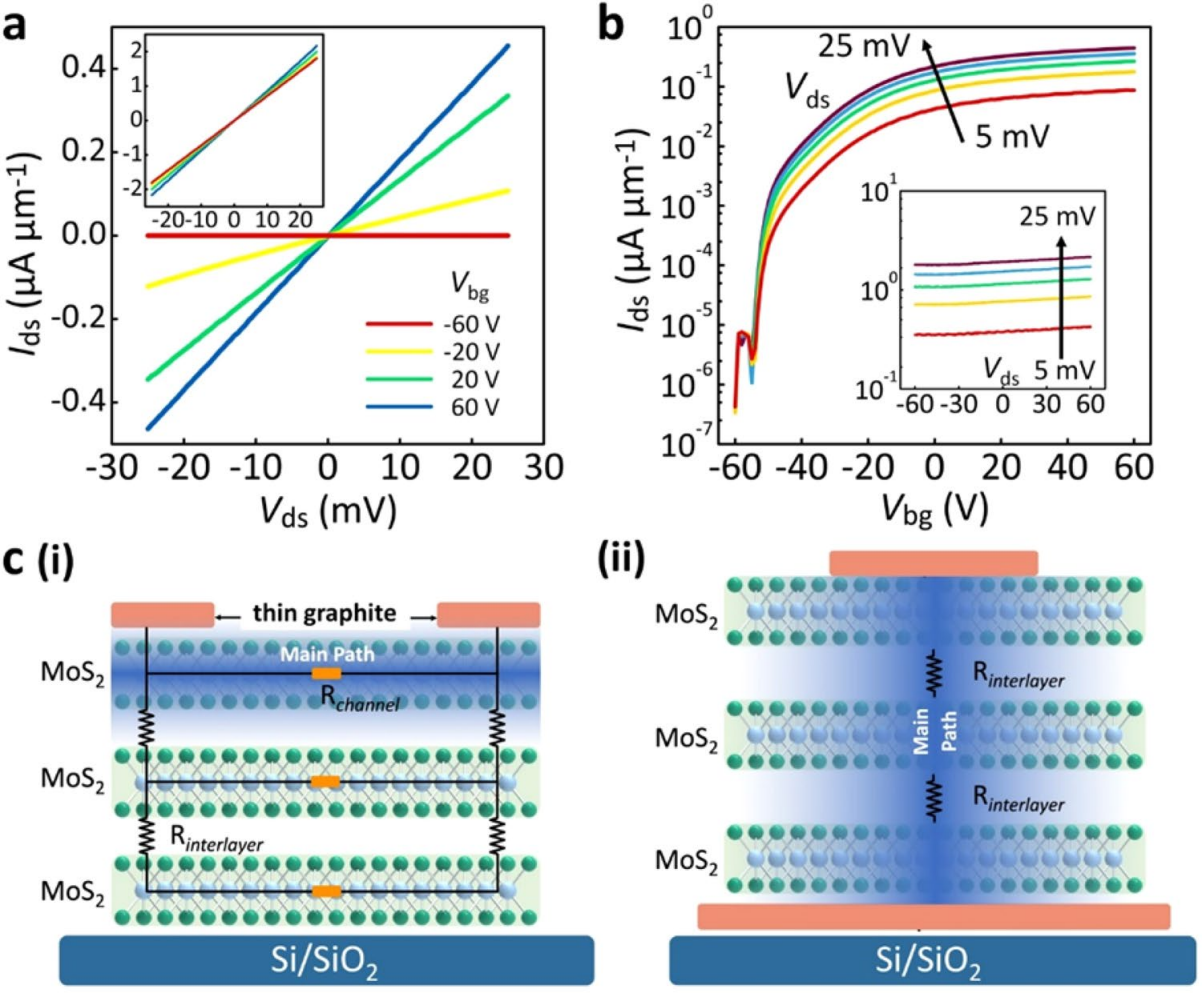
(**a**) *I*_ds_ − *V*_ds_ curves for parallel and vertical (inset) conducting channels at various *V*_bg_ values from −60 V to 60 V. (**b**) *I*_ds_ − *V*_bg_ curves for parallel and vertical (inset) conducting channels at various *V*_ds_ values from 5 mV to 25 mV. (**c**) Schematic illustration of charge transport for parallel (i) and vertical (ii) conducting channels of MoS_2_ electronics, while the shading denotes the main path.

*I*_ds_ − *V*_ds_ curves for both parallel and vertical electronics were examined at room temperature and are shown in Fig. 2(a) and its inset, respectively. The observed linear features of the *I*_ds_ – *V*_ds_ curves in both parallel and vertical electronics strongly imply that contact resistance at the metal-semiconductor interfaces can be neglected, allowing electrical properties as well as carrier transport to be attributed mainly to the MoS_2_ conducting channel. It is emphasized that the contact resistance brings out by the Y-function method to be 3.4 × 10^4^ Ω/μm, which is much smaller than the total resistance of 6.25 × 10^5^ Ω/μm. Injected carriers can easily pass through from one thin graphite electrode to the other due to the formation of the Ohmic contact between the electrodes and the layered MoS_2_ channel. It should be emphasized that the adoption of a four-probe method to double-check the behavior of the Ohmic contact is too difficult to be realized because of the use of mechanical exfoliation. *I*_ds_ − *V*_bg_ relationships for the electronics under different *V*_ds_ values are further provided in Fig. 2(b) on a logarithmic scale. Under modulation of *V*_bg_ values, the parallel MoS_2_ electronics exhibit n-type behavior. The current on/off ratio at *V*_ds_ = 25 mV can reach ~10^6^, which fully matches the requirements for use as a switch. In striking contrast, the vertical MoS_2_ electronics were characterized at room temperature by almost *V*_bg_-independent *I*_ds_ variation, or a slight increase with the increase of *V*_bg_. In addition, because the ~6 nm vertical conducting channel is much shorter than the 1.5 μm parallel channel, the current density in the vertical channel is significantly larger by 1–2 orders of magnitude at room temperature. Unlike the use of parallel electronics for switching, such *I*_ds_ variations in vertical electronics depend only on *V*_ds_ rather than *V*_bg_ (shown in the inset of Fig. 2(b)), and thus show potential for future low-range amplification electronics. In comparison with the previous reports for similar vertical electronics^16,28^, such the weak *V*_bg_ dependence is attributed the absence of tunable Schottky barriers on the contacts. For easy comparison of device performances, effective carrier mobility (*μ*) and subthreshold swing (*SS*) were respectively estimated using conventional equations $\mu =(d{I}_{ds}/d{V}_{bg})\cdot (L/{C}_{ox}\cdot W\cdot {V}_{ds})$ and $SS=d{V}_{bg}/d(\log \,{I}_{ds})$,^29^ where *L* and *W* are the channel length and width, respectively, which has been determined by its optical images. *C*_ox_ of 1.15 × 10^−8^ F∙cm^−2^ is the capacitance per unit area between the MoS_2_ channel and the gate electrode. Notice that although the equation for carrier effective mobility is built for planar transistors, the value estimated from the vertical electronics can be taken as a direct and easy comparison for device performances in between. Respective carrier mobilities for parallel and vertical electronics at room temperature were about 37.06 and 0.31 cm^2^∙V^−1^∙s^−1^. Such lower effective mobility is extracted in the vertical MoS_2_ electronics, suggesting the existence of interlayer resistance between adjacent MoS_2_ layers in the vertical conducting channel. In this channel, carriers have to consume more energy for charge transport, leading to a redundant voltage drop as well as scattering. Although the vertical channel is much shorter than the parallel channel, the electrical contribution of interlayer resistance still significantly restricts *I*_ds_ variations, dominating the transport mechanism. Respective *SS* values of ~1.21 and ~1000 V/decade were estimated for parallel and vertical electronics. The unreasonably large *SS* value for the vertical electronics, consistent with observations seen in the *I*_ds_ − *V*_bg_ curves (Fig. 2(b) inset), again indicates that the inherent contribution from interlayer resistance causes inevitable charge transport scattering and weakens electrostatic control. The performance difference between the two device configurations provides a valuable perspective for the development of complex, stacked 2D electronics.

Figure 2(c) illustrates the corresponding transport mechanisms for (i) parallel and (ii) vertical electronics. The blue areas denote the main path of current flow in the conducting channel, while its color shading denotes the intensity thereof. Charge transport in the parallel channel can be explained by a resistor network model based on Thomas-Fermi charge screening and interlayer coupling^30^. A charge distribution exists in the layered MoS_2_ channel, and the bulk of the charge distribution in the parallel electronics moves to the top layers with increasing *V*_bg_. In on-current states (as shown in part (i) of Fig. 2(c)), carriers are transported mostly on the top and mutual-parallel layers of MoS_2_ channels. Thus, interlayer resistance does not dominate the transport mechanism, allowing layered electronics to represent their intrinsic electrical properties. As for the vertical electronics, carriers must substantially overcome the obstruction of interlayer resistance, resulting in device performance reduction, as shown in part (ii) of Fig. 2(c).

### Temperature dependent electrical properties

**Figure 3:**
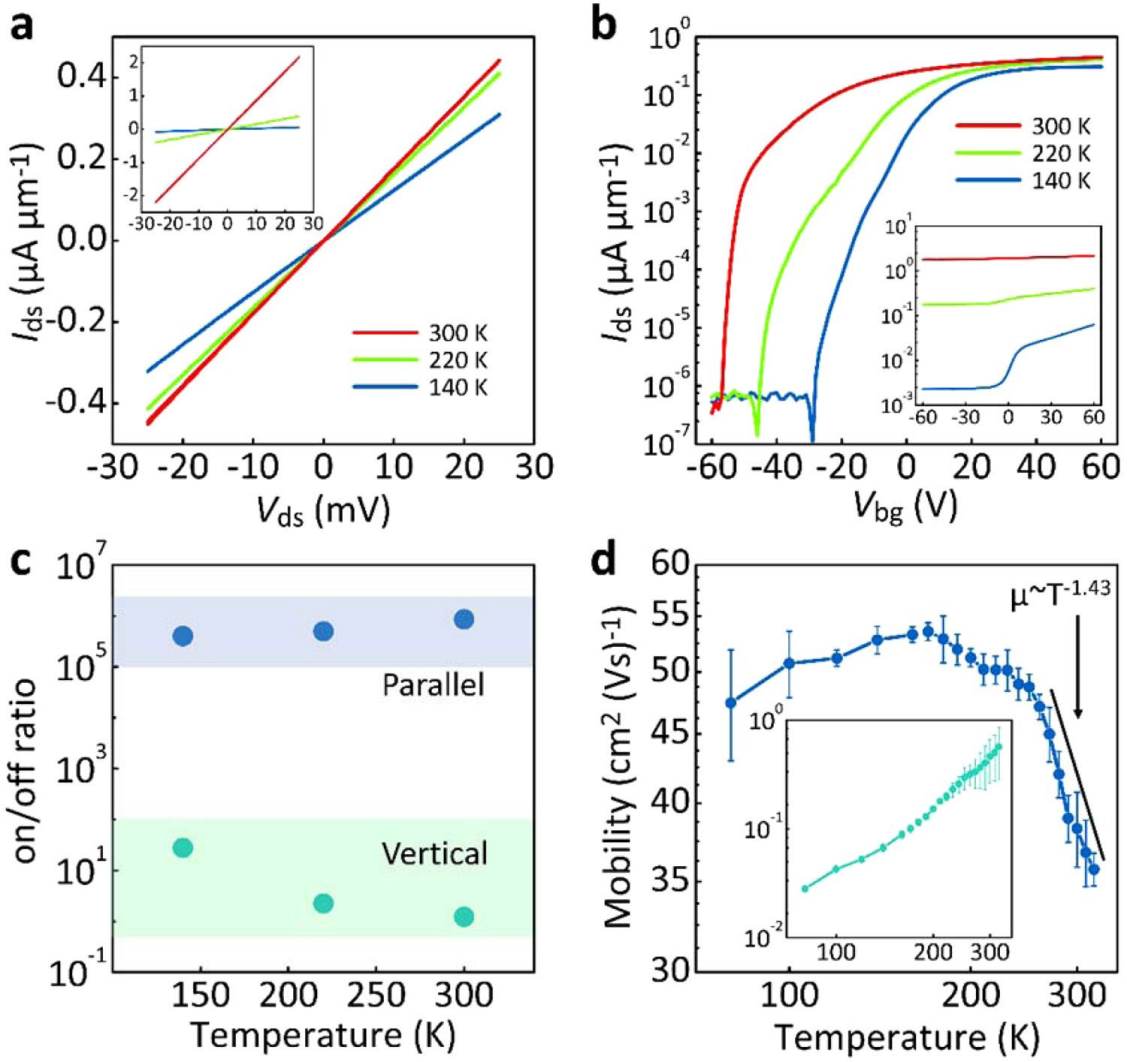
(**a**) *I*_ds_ − *V*_ds_ curves at different temperatures for parallel and vertical (inset) conducting channels at *V*_bg_ = 60 V. (**b**) *I*_ds_ − *V*_bg_ curves for parallel and vertical (inset) conducting channels at *V*_ds_ = 25 mV. (**c**) Current on/off modulation as a function of temperature for parallel and vertical conducting channels. (**d**) Temperature-dependent mobilities for parallel and vertical (inset) conducting channels.

Analysis of *I*_ds_ − *V*_ds_ characteristics of electronics at room temperature alone cannot provide a complete understanding of charge transport or elucidate the microscopic nature of inherent resistance formation^31,32^. Therefore, investigations of temperature dependence were also carried out. Figure 3(a,b) reveal *I*_ds_ − *V*_ds_ and *I*_ds_ − *V*_bg_ behaviors at different temperatures for both electronics in different configurations. With decreasing temperature, the *I*_ds_ − *V*_ds_ curves for both electronics maintain linear form, again implying the formation of an ignored contribution of Schottky barrier at the thin graphite-MoS_2_ interface. Because of the semiconducting nature of the MoS_2_ channel, *I*_ds_ values at a fixed *V*_bg_ and *V*_ds_ for both electronics gradually decrease with decreasing temperature. As for *I*_ds_ − *V*_bg_ behaviors (Fig. 3(b)), the current on/off ratio for parallel electronics indicates weak temperature dependence, consistent with the previous report^33^. On the other hand, the current on/off ratio for vertical electronics increases with decreasing temperature. At 100 K, the current on/off modulation can even reach ~10^2^ for the vertical electronics (see Fig. 3(c)). To provide further insight into the performance difference of both electronics, temperature-dependent mobilities were extracted from 300 K down to 80 K (Fig. 3(d)). It has to be emphasized again such the estimation in mobilitis can offer an opportunity for rough comparison in device performance for both electronics. For parallel electronics, the temperature dependence was characterized by a maximum value around 200–180 K. Above 200 K, a strong decrease in mobility from the maximum value of ~54 cm^2^∙V^−1^∙s^−1^ was observed, attributable to the domination of electron-phonon scattering at higher temperatures. This part of the mobility trend was further fitted by the $\mu  \sim {T}^{-\gamma }$ formula, where the exponent depends on the dominant phonon scattering mechanism. From the fit, the value of $\gamma  \sim 1.43$ is in high agreement with theoretical predictions for a layered MoS_2_ channel^34^. Below 180 K, we found a decrease in mobility, consistent with transport being limited by scattering from charged impurities^35^. As for the vertical MoS_2_ electronics, mobility drastically decreased with decreasing temperature, due to presumable interlayer resistance in the conducting paths. This behavior is akin to the formation of potential barriers between adjacent MoS_2_ layers, which increases charge scattering. At higher temperatures, carriers with higher energy can easily surpass these barriers and exhibit higher mobility. Based on the present evidence, we conclude that, in parallel MoS_2_ electronics, charge transport is dominated by the layered, in-plane 2D channel, which causes effective utilization of 2D material advantages.

### Low-frequency noise

**Figure 4:**
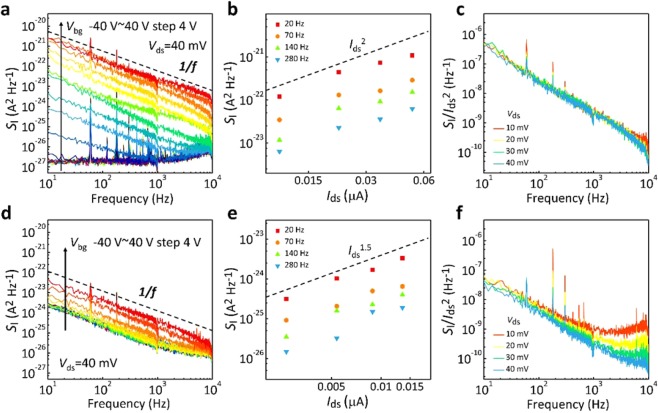
Power spectral densities of current fluctuation (*S*_I_) for (**a**) parallel and (**d**) vertical conducting channels as a function of frequency at 100 K. Dashed lines in (**a**,**d**) denote ideal 1/*f* dependence. (**b**,**e**) *S*_I_ as a function of *I*_ds_ at different frequencies for (**b**) parallel and (**e**) vertical conducting channels. Dashed lines in (**b**,**e**) denote the relationship ${S}_{I}\propto {I}_{ds}^{\alpha }$. (**c**,**f**) *S*_I_ values normalized by the square of *I*_ds_ at different *V*_ds_ values for (**c**) parallel and (**f**) vertical conducting channels.

To go further in characterizing charge transport, we turned to low-frequency (LF) noise measurements using a Programmable Point-Probe Noise Measuring System (3PNMS) with a noise floor ≈ 1 × 10^27^ A^2^⋅Hz^−1^. These measurements, traditionally used in exploring dynamic carrier fluctuations in silicon-based field effect transistors^36–38^, have been widely adopted as a sensitive tool for identifying the influence of interface conditions, particularly for nanoscale electronics. To better understand the differences in transport mechanisms between parallel and vertical MoS_2_ electronics, we conducted LF noise measurements for both parallel and vertical electronics (Fig. 4). For parallel and vertical MoS_2_ electronics, respectively, typical power spectral densities of current fluctuation, *S*_I_, are shown in Fig. 4(a,b), as a function of the frequency at different applied *V*_bg_. The *V*_ds_ value was set at 40 mV while the frequency was swept from 10^1^ to 10^4^ Hz. It should be noted that to effectively reduce the contribution of thermal energy, the measurement temperature was kept at 100 K and monitored. The dashed line, showing an ideal 1/*f* dependence, is provided for comparison. Because of the obvious current on/off modulation, the *S*_I_ for the parallel electronics can be significantly increased, with increasing *V*_bg_, from the system noise floor of 1 × 10^−27^ up to 10^−21^ A^2^∙Hz^−1^ at 10 Hz. This behavior is consistent with *I*_ds_ − *V*_bg_ observations shown in Fig. 3(b). The LF noise data were then analyzed using the formula ${S}_{I}\propto {I}_{ds}^{\alpha }/{f}^{\beta }$, where *α* and *β* are the exponent parameters for current and frequency, respectively^39,40^. Analytical values of *β* for both electronics approached unity closely, indicating the uniform distribution, in energy and space, of intrinsic structural defects such as sulfur vacancies in the conducting MoS_2_ channels. These defects lead to trapping/detrapping fluctuations of current flow. In contrast, analytical values of *α* differed between parallel (~2) and vertical (~1.5) electronics. The evidence used in determining the *α* values is shown in Fig. 4(b,c), where the *S*_I_ value varies as a function of *I*_ds_ across different frequencies. Such deviation from *α* = 2 in the vertical conducting path strongly implies that the existence of an additional resistance contribution incurs energy loss. As mentioned in the above discussion, thin graphite was used in the present study to form a free contact barrier at the thin graphite-MoS_2_ interface. Therefore, this additional contribution originates mainly from interlayer resistances. The *S*_I_ value was further normalized by the square of the *I*_ds_ value at different *V*_ds_ values, as shown in Fig. 4(c,f) for parallel and vertical electronics, respectively. These plots clearly indicate that normalized *S*_I_ cannot collapse into a single curve for the vertical electronics; this finding is consistent with the domination of LF noise by an additional resistor^41^, compared with LF noise in parallel electronics. By determining dynamic current fluctuations, LF noise expresses the difference between two-configuration electronics configurations while addressing the influence of interlayer resistance on charge transport.

## Discussion

In conclusion, the vertical electronics displays almost *V*_bg_ independent *I*_ds_ variation and lower mobility at room temperature, due to interlayer resistance between adjacent MoS_2_ layers in the vertical conducting channel. With decreasing temperature, mobility decreases from ~100 at 300 K down to ~10^−2^ at 100 K. Moreover, LF noise measurements in the vertical channel are consistent with an interlayer resistance contribution. Our study significantly provides fundamental information about electrical properties in layered 2D systems that will likely play an important role in the building of future electronics components as well as van der Waals stacking 3D artificial structures.
